# Predicting the Transition From Depression to Suicidal Ideation Using Facebook Data Among Indian-Bangladeshi Individuals: Protocol for a Cohort Study

**DOI:** 10.2196/55511

**Published:** 2024-10-07

**Authors:** Manoshi Das Turjo, Khushboo Suchit Mundada, Nuzhat Jabeen Haque, Nova Ahmed

**Affiliations:** 1 North South University Dhaka Bangladesh; 2 Vishwakarma Institute of Technology Pune India

**Keywords:** human-computer interaction, depression, suicidal ideation, mental health, India, Bangladesh, Facebook, major depressive disorder, MDD, 9-item Patient Health Questionnaire, PHQ-9, natural language processing, NLP, machine learning, ML

## Abstract

**Background:**

Suicide stands as a global public health concern with a pronounced impact, especially in low- and middle-income countries, where it remains largely unnoticed as a significant health concern, leading to delays in diagnosis and intervention. South Asia, in particular, has seen limited development in this area of research, and applying existing models from other regions is challenging due to cost constraints and the region’s distinct linguistics and behavior. Social media analysis, notably on platforms such as Facebook (Meta Platforms Inc), offers the potential for detecting major depressive disorder and aiding individuals at risk of suicidal ideation.

**Objective:**

This study primarily focuses on India and Bangladesh, both South Asian countries. It aims to construct a predictive model for suicidal ideation by incorporating unique, unexplored features along with masked content from both public and private Facebook profiles. Moreover, the research aims to fill the existing research gap by addressing the distinct challenges posed by South Asia’s unique behavioral patterns, socioeconomic conditions, and linguistic nuances. Ultimately, this research strives to enhance suicide prevention efforts in the region by offering a cost-effective solution.

**Methods:**

This quantitative research study will gather data through a web-based platform. Initially, participants will be asked a few demographic questions and to complete the 9-item Patient Health Questionnaire assessment. Eligible participants who provide consent will receive an email requesting them to upload a ZIP file of their Facebook data. The study will begin by determining whether Facebook is the primary application for the participants based on their active hours and Facebook use duration. Subsequently, the predictive model will incorporate a wide range of previously unexplored variables, including anonymous postings, and textual analysis features, such as captions, biographic information, group membership, preferred pages, interactions with advertisement content, and search history. The model will also analyze the use of emojis and the types of games participants engage with on Facebook.

**Results:**

The study obtained approval from the scientific review committee on October 2, 2023, and subsequently received institutional review committee ethical clearance on December 8, 2023. Our system is anticipated to automatically detect posts related to depression by analyzing the text and use pattern of the individual with the best accuracy possible. Ultimately, our research aims to have practical utility in identifying individuals who may be at risk of depression or in need of mental health support.

**Conclusions:**

This initiative aims to enhance engagement in suicidal ideation medical care in South Asia to improve health outcomes. It is set to be the first study to consider predicting participants’ primary social application use before analyzing their content to forecast behavior and mental states. The study holds the potential to revolutionize strategies and offer insights for scalable, accessible interventions while maintaining quality through comprehensive Facebook feature analysis.

**International Registered Report Identifier (IRRID):**

DERR1-10.2196/55511

## Introduction

### Background

In Bangladesh and India, low- or middle-income countries (LMICs) of South Asia, the alarmingly high increasing rate of suicides is a dire situation [1]. One of the main reasons for this considerable rate, which is 75.5% [2], is that people going through this condition are not diagnosed early [3]. Stigma and gaps in proper treatment related to such mental health conditions have made this problem more widespread [4]. To help reduce this increasing rate of suicide, an automated and easy-to-access solution is required. This work aims to focus on the early detection of mental health problems in a resource-constrained setting [5].

The conventional approach to diagnosing depression requires patients to complete medical questionnaires and is subjective in nature [4]. Many studies have proven that behavior analysis through social media (SM) is the most convenient and fastest way to make the entire process easier and less time-consuming for patients [3,6-8]. Even by inspecting the behavioral patterns, especially by analyzing the text, it is possible to detect major depressive disorder (MDD) [9]. Studies have proven that identifying posts related to suicide begins with diagnosing depression levels, as suicidal ideation is particularly linked to MDD [10,11]. Numerous studies have shown that individuals with depression are at higher risk of attempting suicide [3]. Systems for detecting suicidal ideation using natural language processing (NLP) and machine learning (ML) on SM data are not fully suitable for LMICs like India and Bangladesh due to different behavioral patterns and socioeconomic conditions [12-14]. For example, texting style and grammar patterns are different [15]. Some sensor-based devices can detect depression [16], but this solution is not apt for this region [17].

In Bangladesh and India, the rising rate of suicides presents a grave concern, and statistics reveal an alarmingly high increase [18], with a significant portion (75.5%) attributed to late diagnosis [19]. The stigma surrounding mental health issues and gaps in appropriate treatment are at the root of this problem [20]. Notably, discussions on mental health problems remain neglected and a taboo in these communities, leading individuals to express their concerns primarily through SM platforms, such as Facebook (Meta Platforms Inc), rather than sharing them directly with others [21]. Moreover, SM continues to play a pivotal role in shaping communication patterns and societal dynamics. Bangladesh ranks among the top 3 countries for Facebook active user growth [22], and similarly, India currently has one of the largest user bases for Facebook, which is >314.6 million [23]. By focusing specifically on Bangladeshi and Indian users’ Facebook data [24], the study aims to detect posts related to depression and observe the transition to suicidal ideation. This targeted approach within a specific cultural context will offer unique insights and outcomes, setting it apart from previous research efforts in this domain. Therefore, while building upon existing literature, the study seeks to provide valuable contributions by exploring new dimensions and nuances in the detection and understanding of mental health issues on SM platforms.

**Figure 1:**
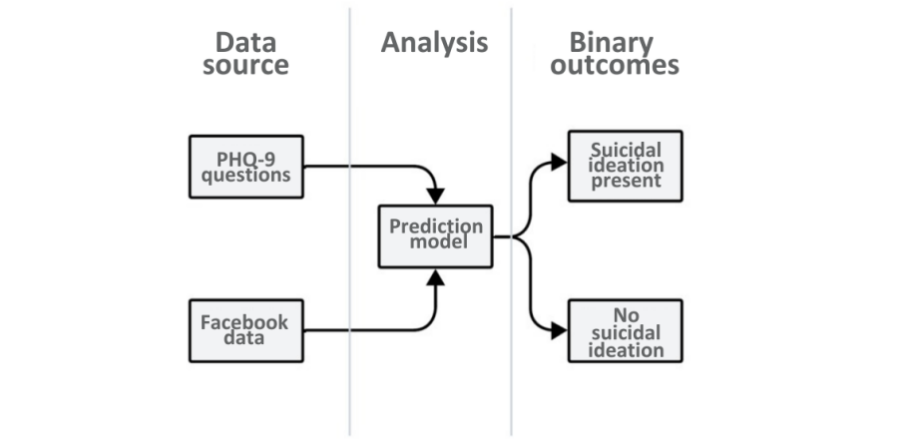
Flowchart demonstrating the steps involved in the suicidal ideation identification system using machine learning (ML) and Facebook data. NLP: natural language processing. PHQ-9: 9-item Patient Health Questionnaire.

We aim to build an automatic system to analyze posts and engagement on SM and predict the person’s behavior and mental state. Moreover, we will use the 9-item Patient Health Questionnaire (PHQ-9) as a validated and reliable patient self-report measure to assess depression [25-27]. We will focus on the interaction and participation of people on SM, which will clearly indicate their interest in communicating their tendencies with others, and text pattern analysis, which will show their emotional state. Most studies have analyzed public account data, but we will also analyze some private account data with users’ consent. We are focusing on some unexplored and unique variables, such as studying engagement with Facebook games and advertisements, analyzing the content of anonymous posts, and establishing a correlation between them and depressive symptoms. In addition, we focus on analyzing data on people from South Asian countries because very few works have been done targeting this region. Moreover, we will ensure the efficiency and usefulness of the model, which we will assess by examining a group of people. In addition, the model will function as a screening tool and symptom-tracking tool, enabling comprehensive assessments of suicidal thoughts, depression severity, and treatment progress as depicted in the Figure 1 [28].

### Related Work

#### Depression and Suicidal Tendency in LMICs

Suicidal ideation, a latent problem, has ranked as the fourth leading cause of death among young adults and adolescents [1]. Close to 800,000 people commit suicide every year [13]. In 2022, Bangladesh reported a total of 446 student suicides [29]. In many LMICs, including India and Bangladesh, suicide rates are alarmingly high, necessitating urgent attention [30]. Depression is a common phenomenon in LMICs due to several factors, including unemployment, poverty, and financial and food crises [31,32]. In LMICs, about one-third of the people commit suicide through the ingestion and inhalation of noxious pesticides; in the Southeast Asian region, 39% of people commit suicide through this method [33,34]. India, in particular, grapples with a substantial number of suicides each year, surpassing 100,000 cases [35]. Notably, suicide ranks as the fourth leading cause of death among young Indians in the 15-29 age group [36]. In Bangladesh, a survey conducted by the Aachol Foundation showed that between January and August 2022, 364 students committed suicide, out of which 194 (53%) were school students and the rest (n=170, 46.7%) were university students [37,38]. Suicide represents a significant social and public health issue that has profound implications for individuals, families, and communities [35]. The causes of suicide are diverse, including professional struggles, social isolation, abuse, family problems, mental disorders, substance addiction, financial challenges, and chronic pain [39]. However, addressing suicide is a complex issue due to various socioeconomic factors prevalent in countries such as India [40] and Bangladesh. Therefore, it is a demanding task to address suicide, which requires multidimensional prevention programs that mirror the multifaceted nature of the issue [4,41].

#### SM Engagement Analysis

People tend to express their emotional state to others. These days, SM has become the main platform [42] where people express themselves through writing. However, engagement on SM varies because of the mental condition of the individual. Studies have shown that people with clinical depression spend more time on SM than people without clinical depression [10,30,43]. By measuring users’ engagement, it is possible to detect their behavioral patterns [44]. To understand behavioral patterns, it is very important to observe emotions such as anger, disgust, fear, happiness, sadness, surprise, shame, and confusion [45]. The observation of the number of daily posts is a fundamental evaluation metric for this project [46], and the details of depressive lexicon analysis will be provided elsewhere. Post segmentation and word representation are 2 approaches that have been used to analyze the results of the depression detection test more effectively. In post segmentation, the sentences are split into words; in word representation, the words are converted into vectors [43]. However, the understanding of how people communicate their feelings through text has not received much attention. After analyzing the premedication and postmedication longitudinal data of a person diagnosed with MDD, it has been clearly stated that the posting pattern has significantly changed the outcome measure, indicating that SM use is affected by a person’s mood [47]. Therefore, an efficient strategy for this study is to concentrate on the text features to understand the user’s mental state and transform them into a quantifiable shape.

Researchers have investigated whether users’ activity and engagement levels right before using Facebook affect how they use it and, if so, then how. They found a noteworthy result from this study: users were affected by the time they spent on SM, and this had a high impact even on their academic performance [48]. However, gathering longitudinal data is a crucial part of this case because these behaviors are entirely dependent on circumstances, which can change very quickly. Modern studies have used the internet and social network information to discover links between individual traits and different disorders. As an example, SM sentiments have been used to predict various factors, such as population happiness [49] and even suicide rates [35,50]. Therefore, it is very important to analyze long-term data to mitigate biases and establish a strong relationship between behavior patterns and quantifying methods.

#### Natural Language Processing

NLP techniques for analyzing Facebook posts encompass a wide range of methods aimed at extracting insights and gaining understanding from text data [51,52]. These techniques include sentiment analysis, which evaluates the emotional tone of posts, enabling the classification of content as positive, negative, or neutral. Named entity recognition identifies and categorizes entities mentioned in posts, such as people, organizations, locations, and dates. Text summarization condenses lengthy posts into shorter, more digestible versions while preserving the main ideas. Topic modeling algorithms, such as latent Dirichlet allocation and nonnegative matrix factorization, uncover underlying themes and patterns within posts, facilitating content organization and categorization [53]. Furthermore, part-of-speech tagging assigns grammatical categories to words, enabling syntactic analysis and the understanding of sentence structures [54]. These NLP techniques empower researchers to gain valuable insights from Facebook posts, ranging from insights into user sentiment and behavior to relevant insights for various applications, such as SM monitoring and user profiling.

#### Text Pattern Analysis

Earlier studies have demonstrated that users openly express signs of depression on SM platforms such as Facebook [55] and Twitter (X Corp) [56]. In certain scenarios, the shared information provides enough insights for researchers to diagnose a major depressive episode. Most studies that have identified depression through SM data are primarily based on the analysis of text from publicly available data. In a particular study, the analysis of Facebook users’ “liked” content unveiled the potential for the precise prediction of diverse traits. Similarly, another investigation revealed that individuals experiencing loneliness are inclined to share more negative content, have fewer friends, and engage in less communication activity on the platform [57]. Valence and arousal, which are the representations of sentiment and intensity, are the 2 most important parameters to examine in any text [58]. Linguistic Inquiry and Word Count (LIWC) is a fully dedicated system for this textual analysis, where it is possible to divide any post into segments and identify the frequently used words, social words, and cognitive processes [59]. Another tool named Natural Language Processing with Java is used to annotate expressions of sentiment and other sociopsychological phenomena using custom lexicons and grammar. It extends the possibilities for the assessment of attitudes expressed in the text beyond sentiment analysis [60]. However, a limitation of these studies is their inability to determine whether the high-frequency words actually indicate suicidal ideation (ie, whether the person has written a post sarcastically).

## Methods

Our procedure will consist of several key steps. These are data collection and preparation, feature engineering, model selection, model training, model evaluation, prediction, and model deployment.

### Data Collection

Our data collection procedure will have 2 parts. The first one is data collection from Facebook, and the second one is data collection with our website, where we will be asking some questions regarding participants’ Facebook use pattern and administering the PHQ-9 to validate our result, which will be predicted using Facebook data.

To streamline the robust and privacy-conscious data collection process for our research, we aim to build a secure web-based system where users can upload their data files [4]. Upon accessing the system, participants would first be directed to a web-based survey where they could provide relevant information and respond to specific questions related to our study. Facebook complies with the General Data Protection Regulation [61] by offering a feature that enables users to download their data, as mandated by the regulation for SM companies. Therefore, following the survey’s completion, participants would be guided to download their Facebook data files directly from the Facebook platform. Further, they were instructed to securely upload the data files to our web-based system for conducting a comprehensive and informed analysis of suicidal thoughts.

Privacy is a multidimensional concept that goes beyond a binary value [62]. Therefore, it is essential to respect individuals’ autonomy [63] and data control, making it inappropriate to ask users to make their accounts public for research purposes. For individuals facing suicidal thoughts, privacy is crucial [64,65] in fostering a supportive and safe environment, allowing them to seek help without the fear of stigma or reluctance [66,67]. Respecting privacy in research is crucial to upholding ethical standards and protecting participants’ personal information [64]. Therefore, we will refrain from asking participants to make their accounts public to uphold the principles of privacy and respect individuals’ confidentiality in our research.

While Facebook restricts external entities from accessing its data [68], authorized application programming interfaces (APIs) such as the Facebook Graph API [69] enable the retrieval of publicly available information about Facebook users while adhering to privacy and legal guidelines. However, the API comes with several limitations and concerns [70,71] that can introduce biases into the model for predicting suicidal ideation, affecting the accuracy and effectiveness of the predictions.

### Clinical Assessment

To diagonalize and comprehend the severity of depression, we will use the PHQ-9. This questionnaire comprises 9 questions with a scoring range of 0 to 27. The questionnaire categorizes depression into 5 segments: 0 to 4 as minimal, 5 to 9 as mild, 10 to 14 as moderate, 15 to 19 as moderately severe, and 20 to 27 as severe. The ninth item, “Thoughts that you would be better off dead or of hurting yourself in some way,” directly indicates users’ perspective on suicidal thoughts. This assessment is an important point to focus on to understand the shift.

### Ethical Considerations

This study has undergone a comprehensive human participant research ethics review and has obtained the necessary approvals. Approval was granted by the scientific review committee from North South University, Dhaka, Bangladesh, on October 2, 2023 (NonCTRG-23-38), followed by institutional review committee ethical clearance on December 8, 2023 (2023/OR-NSU /IRB/1033). Informed consent was diligently obtained from all participants involved in the study, ensuring transparency and ethical integrity. Moreover, stringent measures were implemented to anonymize and deidentify study data, thereby safeguarding the privacy and confidentiality of human participants involved in the research. These ethical considerations underscore our commitment to upholding the highest standards of research integrity and participant welfare throughout the study process.

### Participants

The studies investigating the prevalence of suicidal ideation outcomes among adolescents or young adults are dependent on factors such as age, gender, socioeconomic status, and cultural differences in India [72] and Bangladesh [73], as well as factors such as the frequency and intensity of SM use, types of content shared, and the presence of mental health indicators in web-based behavior. Given the complexity of the study and the need for robust statistical analysis, we anticipate determining the sample size based on a power analysis approach and earlier studies conducted in similar settings. We have carefully considered previous research approaches and sample sizes. The study by Patel et al [74] used larger sample sizes, ranging from 1500 to 30,000 participants, allowing for comprehensive analyses and insightful conclusions. Conversely, smaller sample sizes, ranging from 100 to 500 participants, were used in studies such as those by Vornholt and De Choudhury [75] and De Choudhury et al [76], demonstrating the feasibility of conducting impactful research even with fewer participants.

Given the diverse populations and SM use patterns in India and Bangladesh, we have tailored our sample size determination strategy accordingly. While these countries have millions of inhabitants [77,78], the subset that actively engages with SM, particularly Facebook, and is willing to share personal data for research purposes is smaller. In addition, to ensure balanced representation, we will carefully select participants to achieve a proportionate distribution of positive and negative samples once the minimum threshold value is reached. It is worth noting that despite India’s larger population, our study anticipates a greater participation rate from Bangladesh due to higher Facebook use. Therefore, we aim to recruit 300 to 1500 participants from Bangladesh and 300 to 700 participants from India. This approach ensures a sufficient sample size to yield meaningful insights while considering the unique demographic and SM landscape of each country.

### Text and Behavior Analysis

#### Overview

We are planning to use NLP techniques to analyze the linguistic patterns and sentiments in user-generated content. We will also identify language indicative of depressive symptoms, negative emotions, and expressions of hopelessness. We will use LIWC and Affective Norms for English Words lexicon categorization for more accurate results in the text analysis [79]. In addition, we will use topic modeling and keyword extraction to identify prevalent themes within the text. These insights are essential for understanding trends, preferences, or concerns within the data set.

Behavior has 2 divisions: post-centric behavior and user-centric behavior. Post-centric behaviors include post time, number of posts, posting frequency, friends, followers, likes, replies, comments, and emoji counts. User-centric behaviors include positive affect, negative affect, activation, dominance, linguistic style, depression lexicon, and antidepressant use. Linguistic style specifically shows the total word count, first-person and third-person pronouns, functional words, assent, negation, etc.

#### Facebook Game Analysis

According to studies, people who play video games excessively are more likely to experience sadness and social anxiety [80]. Our observation supports this assertion that the number of people playing Facebook games grew dramatically throughout the COVID-19 era [55].

A gaming function on Facebook offers a variety of games that can be played by one person or several people. The accompanying player does not necessarily need to be on the friend list of the user but can be anyone from the Facebook community. A few well-known games are carrom, Bubble Shooter, Uno, Ludo, and chess. People can challenge one another in the game, and if the other person is interested, they can accept the challenge and finish the game. Most likely, these games are web based and can be played directly from a browser without downloading, but some of them also offer an Android version that needs to be downloaded on the phone.

A few factors to consider while analyzing gaming are frequency, duration, genres, single-player versus multiplayer, and in-game behavior.

There are currently no set standards for identifying addictive and nonaddictive players. However, there is a strong relationship among weekly gaming time (WGT), craving, and the problem video game playing (PVP) score. Hard-core or even excessive players (extremely high WGT and PVP scores) as well as nonexcessive players (low WGT and PVP scores) show distinct patterns in their gameplay behavior [56]. It has been established that negative emotional ratings for excessive gamers are higher than for nonexcessive players. In addition, they also have higher “self-devaluation when facing failure” scores. The excessive players displayed higher negative feelings and negative bodily manifestations in terms of both emotions and physical symptoms. This seems to be in line with the idea of addiction, when the action no longer produces happy results but instead causes negative ones. Playing video games over a long period might cause a disorder called alexithymia, which impairs the capacity to recognize one’s own internal emotions. People’s inability to identify particular emotions contributing to their current state of mind might make it worse [81]. Due to the greater focus and cognitive processing required by skill games, these players favor action games over them [82].

Games are categorized based on their intensity level: casual games are simple and easy-to-play games designed for short play sessions, whereas hard-core games are more complex and require a higher level of skill and commitment [50].

Categorizing games according to intensity level, we distinguish between hard-core and casual categories. The hard-core category includes games such as Magic Swap Puzzle, Coin Match, Solitaire, Words With Friends, Quiz Planet, Krunker FRVR, Uno, Chess, and Word Search. In contrast, the casual category comprises games such as Bubble Shooter Pro, Bubble Pop, Basketball FRVR, Ludo Club, Cooking Trendy, Carrom Board, Card Party, Card Wars, Eight Ball Pool, and The Test. This distinction helps in understanding player engagement and preferences based on the intensity of gameplay.

#### Description Analysis

In the bio section, people generally write about themselves or any famous quote they like or that resembles them. Sometimes, it can be one or multiple lines or even just a word. In the details section, they can add current and previous workplaces, institutions, relationship status, hobbies, and cities they previously lived in and currently live in. People with mental health issues have a tendency not to share their information with everyone. They have a close group of people with whom they feel comfortable sharing information [57]. It is often seen that the bio and details sections of these people remain almost empty or filled with very little information.

#### Content Analysis

Studies have consistently shown that people are willing to share their depression and medical conditions on various social networking sites [83]. Related studies have investigated language and emotional patterns and successfully assessed new mothers’ postpartum behavioral changes from prenatal assessments [84]. These findings highlight the potential of SM as an important indicator in assessing the occurrence of current or future depression.

In our study, we aim to introduce measures that evaluate and characterize users’ linguistic styles in posts, comments, and search history, irrespective of whether they are anonymous. An important observation is that users who are depressed post personal content in various groups or pages anonymously. This behavior seems driven by the need for a safe, nonjudgmental space to express emotions and seek support. In addition, depression-related content, such as quotes, internet memes, and music, is prevalent among users. Examples include posts including “sad songs” and “mental illness quotes,” along with expressions of feelings of despair and overwhelm, such as “I am so lonely” and “Everything seems too overwhelming and pointless.” Understanding such patterns of anonymously shared data on SM platforms can provide valuable insights into predicting the mental health challenges people face. Moreover, analyzing such content and the frequency of posting may be instrumental in identifying depression and the potential areas for targeted interventions and support.

This study also highlighted the untapped opportunity in understanding users’ search activity, which holds promising potential for enhancing clinical and public health efforts in suicide risk assessment and prevention. Participants’ search themes encompass suicide, help-seeking, mood and anxiety symptoms, and trauma and negative life events [85]. Often, users facing trauma or negative life events search for support resources, coping strategies, and information on seeking professional help. This includes asking questions about outpatient resources, health clinics, and other medically relevant topics, analyzing which helps gain a comprehensive understanding of users’ mental health concerns. Further, to enhance the robustness of the predictive model, in this study, we focus on additional insights into users' engagement with relevant resources and communities over Facebook. By integrating a comprehensive list of the user’s group memberships, liked pages, and notification messages, we provide a holistic approach to understanding users’ mental health, allowing us to develop effective strategies while assessing suicide risk at an early stage.

In this research paper, we will preprocess the text data to ensure their suitability for subsequent model building. Given the unstructured nature of SM data, we will apply customized preprocessing and cleaning techniques. These techniques include removing accented characters; expanding contractions; converting to lowercase; eliminating URLs, symbols, digits, and special characters; fixing word lengthening; applying spelling correction; removing stop words; and performing lemmatization. These steps will aim to standardize the text, reduce dimensionality, improve data quality, and ensure consistent representation in our models. By implementing these preprocessing steps, we will be able to successfully clean and prepare the text data, making them suitable for subsequent analysis and model training [74].

During the data cleaning phase, we will take additional steps to refine the preprocessed text data. These steps will involve removing irrelevant words, empty rows, and outlier rows with high word counts. By eliminating irrelevant words, we will focus on more meaningful features for analysis. The empty rows, which will not contain any useful information, will be dropped to ensure data integrity. At the same time, the outlier rows with high word counts, deviating significantly from the majority, will be removed to optimize model training efficiency. These measures will improve the data set’s quality and relevance, preparing it for further analysis and model training.

Further, we will use a vectorizer called Tf-idf Vectorizer to transform our textual data into numerical features suitable for ML algorithms. That vectorizer will can be configured with specific parameters: min_df=50 and max_features=5000. The min_df parameter specifies that a word must appear in at least 50 documents (posts, in our case) to be included in the feature representation. This will help filter out infrequently occurring words that may not contribute significantly to the overall understanding of the data. The max_features parameter sets an upper limit on the number of features (words) to be considered. In our case, we will set it to 5000, indicating that only the top 5000 most relevant features (based on their frequency) will be selected for further analysis. This approach will enable us to represent the text data numerically, capturing the importance of different words while reducing the dimensionality of the feature space.

#### Diurnal Activity

Diurnal activity indicates the activeness of a person on SM both during the daytime and at night. It has been mentioned that individuals who are depressed are most active during the night [6,30,86]. In our study, we will investigate the moods [87] of all people who have written posts on SM. We intend to determine the emotions of a person who is depressed (ie, to visualize whether the person is actually depressed).

Diurnal activity will be calculated using the following formula:

XG(d,h) / ∑XG(d,i) **(1)**

where XG (d,h) indicates the average timing of the total number of posts made in hours (h) and days (d), the G in XG indicates the country where the post was made. In addition, h indicates a value from 0 to 23 (ie, an average number of posts is considered up to 11 PM).

Another study indicates a positive relationship between the use of emojis and genders (ie, women use more emojis than men), and the most frequently used emoji was the emoji representing tears of joy, with a use percentage of 22.2% and 18.9% among women and men, respectively. However, to investigate the emoji choices of both genders, a statistical analysis was carried out, named “mutual Information” (MI), which calculates the use of emojis by both men and women [88]:

MI(X;Y)e = ∑∑P(x,y)e log (P(x,y)e) / (P(x)e × P(y)e) **(2)**

where X indicates whether the gender (either men or women) uses an emoji. Here, the value of x will be equal to 1 if a user applies emojis, and if the value of x is equal to 0, then the user does not apply emojis. However, Y indicates a user’s gender (ie, for men, the value of y will be equal to 1; for women, the value of y will be equal to 1). Here, X is represented as a member of x, and Y is represented as a member of y. In contrast, p(x,y)e indicates the joint probability of X and Y, whereas p(x)e and p(y)e indicate the marginal probability of X and Y [88].

For analyzing the data from Facebook, we will observe the time stamps for negative posts and the types of emojis that are used frequently on posts related depression. We will be focusing on the formulas given earlier, but we will adjust the variables to suit our needs.

For “negative” emotions from posts for “diurnal activity,” we will be applying the text2emotion Python function to extract the emotional words used on the posts. However, the text2emotion library shows 5 different emotions, namely happy, sad, angry, surprise, and fear. However, here, we will be dealing with the negative feelings from Facebook posts.

First, our main purpose is to clean the data to make them appropriate for analyzing emotions. For the data cleaning process, we need to follow several steps. For instance, we need to remove the unwanted texts or words from the posts (ie, the words that have low frequency based on term frequency–inverse document frequency), apply various tools of the Natural Language Toolkit for sentiment analysis, and extract the better-preprocessed posts from the previous preprocessed posts. Second, we need to figure out the emotional words from the preprocessed texts and then use them for further visualization of the emotions. Finally, we need to observe the scores of the emotions. The higher the score from 1 of the 5 categories, the more the emotion will belong to that group.

After that, we intend to observe the emojis from the posts to investigate what type of nonverbal attributes a person who is depressed has both during the daytime and nighttime while writing posts on SM. This will help us figure out which emojis a person who is depressed uses repeatedly during the night. Several studies were conducted on emojis [89-92], indicating how they coincide with the linguistic style of a person. For instance, the emojis that are inclined with the negative words from LIWC are “anger,” “money,” “ingest,” “family,” “home,” and “death” [89].

As mentioned earlier, we will be focusing on the sentiments in the posts and the frequently used emojis by an individual during nighttime. Moreover, we will be considering how the emotions in a person’s post and the number of emojis used on a post are correlated with diurnal activity. For that, we will be using the formula for MI, as it consists of both joint probability and marginal probability. “Joint probability” will help investigate the relationship between the emotions in the posts and the number of emojis used, applying a condition, that is, if we work on the sentiments in the posts, then the number of emojis will be kept constant, and vice versa. In contrast, “marginal probability” will help us extract 1 feature at a time (ie, the features will be independent of one another).

There will be a change in the formula for both *“*diurnal activity*”* and MI (ie, in the *“*diurnal activity” formula, the number of posts will be denoted as “Po,” and not XG). For MI, instead of “X” and “Y,” we will write “Po” and “E,” respectively. Here, “Po” will indicate the posts written by an individual during nighttime, and “E” will indicate the types of emojis used by that individual.

The modified formulas for diurnal activity and MI are as follows:

(Po(d,h)) / (∑Po(d,i)) **(3)**

MI(Po,E) = ∑∑P(Po,E)e log (P(Po,E)e) / (P(Po)e × P(E)e) **(4)**

To find out the MI, we will consider various combinations for the number of “negative” posts and emojis used by an individual. We will consider the values of both Po and E as binary 1 (ie, Po=1 and E=1) if the posts are “negative” and the emojis are used.

In contrast, we will consider the values of both Po and E as binary 0 (ie, Po=0 and E=0) if the posts are “nonnegative” and no emojis are used in the post.

There are other combinations as well, that is, Po=0 and E=1 if the number of posts is “nonnegative” and emojis are applied in the posts, and Po=1 and E=0 if the posts are “negative” and no emojis are used in the posts.

Here, we will eliminate Po=0 and E=1 and Po=0 and E=0, as we will not deal with “nonnegative” posts (ie, Po=0) and “zero emojis” (ie, E=0).

The highest value of MI will indicate both the number of “negative” posts and “emojis” (Po=1 and E=1) used. In contrast, the lowest value will indicate either the number of “nonnegative” posts and “emojis” (Po=0 and E=1) used or the number of “nonnegative” posts and “zero emojis” (Po=0 and E=0).

#### Advertisement Analysis

Participants revealed a tendency to reduce their SM posts during periods of low moods, with the belief that their SM content might not accurately depict their depressive state. SM platforms such as Facebook use algorithms to direct advertisements to suitable users, leveraging search keywords and previously accessed links. A subset of participants acknowledged the practice of targeted advertising, while a minority reported posting specifically to seek support. In addition, participants acknowledged the existing use of targeted advertising and expressed a preference for its application in mental health care provisioning as opposed to its current use. Individuals experiencing depression tend to conduct searches related to health-related topics, resulting in personalized advertisements that align with their search preferences.

#### Network Analysis

For measuring the network, we will calculate reciprocity, cluster coefficient, and betweenness centrality. This will help understand the user’s close connections and how frequently they communicate with them. To analyze the post-centric and user-centric behavior in detail, we need to calculate reciprocity, prestige ratio, clustering coefficient, degree centrality, closeness centrality, betweenness centrality, and eigenvector centrality.

In terms of analyzing the network, we will consider “u” as the user whose network property we will analyze. A node indicates a person, and an edge is considered a connection or link. Reciprocity will reflect how often “u” has responded to any message.

The prestige ratio indicates the bidirectional communication of the user. That means after texting back to any message, it tracks the number of times they have participated and the frequency of their interactions.

Mathematically, the reciprocity formula is as follows:

Reciprocity = (number of reciprocated connections) / (total number of connections) **(5)**

To calculate the reciprocity, we have to count the number of connections between pairs of users in a network and determine how many of these connections are reciprocated. A reciprocated connection occurs when 2 nodes have a mutual connection with each other. For example, if user A is connected to user B and user B is also connected to user A, it is considered a reciprocated connection. A higher reciprocity score in network analysis indicates a more tightly connected and interactive network with stronger bidirectional relationships between users.

Clustering coefficient measures how likely it is for the friends or connections of a user to be friends or connected to each other. Basically, it provides a quantifiable measure of how much users tend to form groups or clusters within the network.

For user i, who has ni neighbors, the clustering coefficient Ci is defined as the ratio of ei connected pairs to the number of all possible connections among the ni neighbors:

Ci = 2ei / (ni([ni – 1]) **(6)**

The formula calculates the clustering coefficient by dividing the number of actual connections between the neighbors of the user i (2ei) by the number of possible connections between the neighbors of the user i (ni([ni–1]).

A higher clustering coefficient score in SM analysis signifies a network with well-connected subgroups or clusters, indicating the presence of close relationships and social interactions among users within these groups. A lower clustering coefficient score in social network analysis indicates a network with less local interconnectedness and fewer cohesive groups, suggesting a more diverse and less tightly knit social structure among the nodes.

Closeness centrality annotates how easily “u” can reach other users in a community. To be more specific, it measures how close or directly connected a user is to other users. A high closeness centrality indicates that “u” is closely connected to many other users. A low closeness centrality indicates that “u” is distant from others in the network. It means “u” may need to go through multiple intermediate connections to reach other users.

Closeness centrality, as the name suggests, is an index defined in terms of distance. Length of a (s, r) path represents the number of edges contained in it. We define the (shortest path) distance, dist (s, r) of s, r∈V as the minimum length of any (s, r) path. We reiterate that we consider only connected graphs for now and observe that dist (s, s)=0 for all s ∈ V. The distance matrix D=(dist[s, r])s, r∈V of an undirected graph is symmetrical, so the total distance, dist(v), of a vertex v ∈ V is obtained as either the row or column sum:



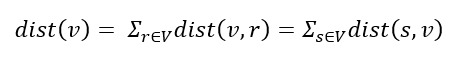



**(7)**

Because of this reversal in ranking, the closeness centrality of a vertex s ∈ V is usually defined as the inverse of the total (or, equivalently, average) distance [49]



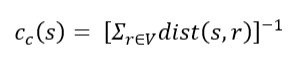



**(8)**

Higher closeness centrality indicates that a user is more central and well connected in the network, while lower closeness centrality suggests that a node is less central and may have less efficient access to other nodes.

Betweenness centrality is a widely used measure that captures a user’s role in allowing information to pass from one part of the network to another. It shows the potential of a user in terms of communication:



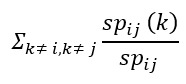



**(9)**

The number of shortest paths from a vertex i to a vertex j that goes through a vertex k, denoted sp_ij_ (k), is the maximum number of shortest paths from vertex i to vertex k in the shortest path tree rooted at i and the number of shortest paths from vertex j to vertex k in the shortest path tree rooted at the vertex j [93].

Users with higher betweenness centrality have more control over the flow of communication in the network and act as important mediators or connectors between different parts of the network. In contrast, users with lower betweenness centrality have less influence over the overall communication patterns and may be more localized in their interactions. Betweenness centrality is a measure of a user’s importance in facilitating communication, and it provides insights into the user’s role in maintaining network connectivity and communication.

### ML Modeling

We are planning to train and validate ML models using labeled data, encompassing a balanced number of posts from individuals with diagnosed depression and those without. After that, we will use a combination of features, including sentiment scores, word frequency, and contextual information. We will conduct an extensive analysis using classification models to predict suicidal risk by assembling multiple classification models based on the provided features. The customized models used in the voting classifier will include random forest, decision tree, gradient boosting, XGboost, and k-nearest neighbors.

We will conduct an extensive analysis using various classification models to predict suicide risk using multiple classification models based on the provided features. These models include a voting classifier, random forest classifier, decision tree classifier, gradient boosting classifier, XGBoost classifier, and k-nearest neighbors classifier. Initially, we will split the data set into training and testing sets with a ratio of 7:3. We will use a voting classifier to combine the predictions of 3 naive Bayes classifiers. In addition, we will optimize the performance of the random forest classifier, decision tree classifier, gradient boosting classifier, XGBoost classifier, and k-nearest neighbors classifier by tuning their hyperparameters using techniques such as RandomizedSearchCV. At the same time, we will consider the characteristics of our data set and the nature of the classification task to choose an appropriate model that can effectively capture the patterns and relationships in the text data. Therefore, we will evaluate the performance of each model by calculating training and testing scores. This comprehensive methodology will allow us to identify the most effective model for predicting suicide risk based on the given features.

One of the potential concerns of this study is addressing the data imbalance challenge, particularly regarding the occurrence of suicide. To mitigate this imbalance, we have studied the techniques that were previously implemented in similar settings. Firstly, we will use appropriate data preprocessing techniques, such as oversampling or undersampling methods, based on the collected data to ensure that the predictive model is trained on a balanced data set. In addition, feature selection methods will be used to prioritize the most informative variables related to depression and suicide prediction, thereby minimizing the impact of data skewness on the model’s performance. Furthermore, we will consider the use of robust evaluation metrics, such as precision, recall, and *F*_1_-score, which will allow for a comprehensive assessment of the model’s predictive capability across different outcome classes, effectively addressing the challenge posed by data imbalance. These approaches would provide valuable insights into addressing data imbalance challenges in predictive modeling studies related to mental health.

### Evaluation Metric

#### Overview

In the process of evaluating our models, we will use various ML metrics that offer critical insights into their performance. These metrics play a pivotal role in determining the accuracy and efficacy of the models. Specifically, we will be focusing on the following metrics [48]: true positive, false negative, true negative, and false positive. These metrics hold significance in assessing the predictive capability of the models in a classification context.

#### Sensitivity (True Positive Rate or Recall)

This metric measures the ability of the model to correctly identify people who are depressed. It calculates the ratio of true positives (correctly identified people who are depressed) to the sum of true positives and false negatives (people who are depressed incorrectly classified as people who are not depressed).

#### Specificity (True Negative Rate)

This metric evaluates the proportion of actual negatives (people who are depressed) that are correctly identified as such. It measures the accuracy of identifying healthy individuals who do not have depression.

#### Precision (Positive Predictive Value)

Precision calculates the fraction of relevant instances (positively labeled) among the predicted instances. It provides an assessment of the accuracy of positive predictions made by the model:

TP / (TP + FP) **(10)**

#### F1-Score

The *F*_1_-score is a measure that combines precision and sensitivity (or recall) into a single metric. It calculates the harmonic mean of precision and sensitivity and is particularly useful when dealing with imbalanced data:

(2* precision score × recall score) **/** (precision score + recall score) **(11)**

#### Accuracy

Accuracy represents the overall performance of the model, measuring the proportion of correctly classified instances out of the total instances. The error rate, which complements accuracy, quantifies the proportion of misclassified instances:

(TP + TN) / (TP + TN + FP + FN) **(12)**

#### Evolution

**Figure 2:**
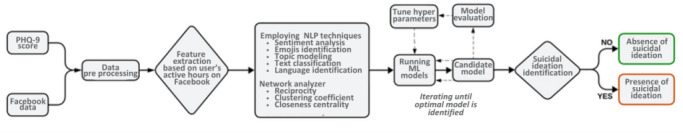
Deployment of the suicide ideation prediction model based on the 9-item Patient Health Questionnaire (PHQ-9) and Facebook data examination. ML: machine learning; NLP: natural language processing.

Among the array of standard evaluation metrics, precision, recall, and *F*_1_-score are given more weightage to assess the efficacy of models in accurately classifying depression classes. When assessing early detection algorithms, it is crucial to consider not only the accuracy of decisions but also the associated delays. In line with this, Wang and Mark [48] introduced the early risk detection error metric, which penalizes untimely correct decisions to address this concern. Flatency, introduced in the CLEF eRisk 2021 [59], is an interpretive evaluation metric proposed alongside the early risk detection error metric to enhance interpretability [30]. In addition, known as latency-weighted *F*_1_, flatency combines *F*_1_-score with speed, reflecting both accuracy and efficiency in identifying users’ content, particularly emphasizing early and accurate classification. By considering these metrics, we can gain a comprehensive understanding of the performance of our models in terms of their ability to correctly identify individuals who are depressed and individuals who are not depressed. The streamlined flow of the depression identification system is depicted in Figure 2.

## Results

Our system is anticipated to automatically detect posts related to depression by analyzing the text and use pattern of the individual with the best accuracy possible. Ultimately, our research aims to have practical utility in identifying individuals who may be at risk of depression or in need of mental health support.

Simultaneously, by analyzing daily activities on SM, we aim to determine how frequently individuals write “negative” posts on Facebook, as such posts are often associated with depression [94]. Additionally, we will examine the types of emojis that are used repeatedly by these individuals. This will help us identify how often an individual uses emojis while writing a post. Moreover, if a person does not use an emoji, their posts will be detected based on the score of their “negative” emotional words (ie, “sad,” “angry,” or “fear”).

By accurately classifying individuals based on their text analysis, we hope to contribute to the development of early detection systems or interventions that can enhance mental health screening and well-being.

## Discussion

### Overview

In this study, we will focus on the South Asian region, mainly Bangladesh and India, which is still dealing with economic issues and some problems with regard to basic rights. People from this region do not pay adequate attention to mental health and well-being. Therefore, we aim to build such a solution that will cover most of the gaps regarding all the mentioned facts. Our goal is to establish a procedure that will analyze how a collection of behavioral indicators that appear on SM may be used to forecast postings that are suggestive of depression and, in turn, comprehend widespread suicidal tendencies in populations.

### Principal Findings

Our study will begin by administering basic questions to users, followed by a filtering process to select suitable participants for our study. Upon obtaining the Facebook data, our analysis will commence. We will initially determine whether Facebook serves as the primary SM platform for the participants, a crucial factor influencing the accuracy of our predictions. In case it is their main platform, we will focus on their interactions, encompassing messages, posts, comments, and gaming activities. Our analysis will extend to anonymous actions and their engagement with content related to depression.

Conversely, in instances where Facebook is not the predominant medium, judicious focus is shifted to the advertisement analysis model. This model hinges on deciphering users’ web-based behavior, scrutinizing their browsing history, visited sites, and thematic inclinations, particularly within realms such as consultancy or support services. For participants who primarily use other platforms, our analysis will shift to evaluating the types of websites they frequent and the content they engage with. This system will work privately to assess the risks associated with anticipated changes in the future while taking into account metrics related to SM activity. We aim to demonstrate a significant correlation among Facebook games, user profile information, and content such as advertisements on mental health as depicted in Textbox 1.

Finally, we will incorporate an assessment of the frequency of their posting, commenting, friend engagement, and previous questionnaire responses, thus enhancing the depth and accuracy of our predictive model. Our ultimate prediction will amalgamate these distinct analyses through advanced techniques. Informed by the outcome of these individual models, our predictive synthesis advances, culminating in a definitive assessment of the presence or absence of suicidal ideation.

**Consideration of data variables for suicidal ideation detection based on Facebook activity status.**

- If a user is active on Facebook
- 9-item Patient Health Questionnaire (PHQ-9) score
- Facebook games analysis
- Bio analysis
- Post and comment analysis with or without the user’s name
- Notification analysis
- Kinds of groups joined and pages liked analysis
- Search history analysis
- Advertisement analysis
- Diurnal activity value
- Network attributes calculation

**If a user is not active on Facebook but has created an account**

- PHQ-9 score
- Advertisement content analysis

### Comparison With Previous Work

Our study will present a novel investigation, first exploring the prevalence of Facebook as the dominant social application, whereas existing studies focused on Twitter, Reddit (Reddit Inc), and Instagram (Meta Platforms Inc) as data sources [95]. We will use a process to collect the data that will not require any extra website or setup to be downloaded or installed on the user’s end. This is the reason why we are claiming our process is easy to access. Other existing solutions need some external infrastructure, which makes the data donation process a bit complex for users. Along with all the engagement analysis parameters, including texting patterns, we will also consider some unique parameters such as used emojis, stickers, Facebook games, bio sections, and advertisement suggestions that have a direct connection with users’ behavior. People with MDD have noticeable difficulties with their attention span [96]. On this account, we are showing a connection between these parameters and users’ behavior. This exploration will provide valuable insights into the emotional state of users.

### Limitations

However, this study may have a few limitations and biases that are crucial to consider. First, the recruitment of participants through web-based platforms may introduce selection bias, as individuals who are active on SM may be different from the general population [97]. In addition, reliance on self-reported data, such as responses to demographic questions and mental health assessments, may introduce response and social desirability biases. Participants may underreport sensitive information or provide socially acceptable responses, leading to inaccuracies in the data collected [98]. Moreover, the interpretation of textual data, including language nuances and sentiment analysis, is subject to inherent biases in NLP algorithms [99]. Future research should aim to mitigate these challenges through methodological refinements and validation studies.

Future research directions can include comprehensive validation studies that directly compare the outcomes of our screening method with those of clinical assessments conducted by mental health professionals. This validation process would involve recruiting control groups and conducting direct comparisons to assess the accuracy and reliability of our screening results in identifying individuals with suicidal ideation. This will provide valuable insights into the strengths and limitations of our approach. By establishing the concordance between our screening results and clinical diagnoses, we can enhance the credibility and utility of our screening methodology in real-world settings [100]. Researchers can also conduct a broader exploration of alternative screening methods and interventions based on the screening results. Incorporating additional data sources or using advanced ML techniques could enhance accuracy and efficiency. In addition, developing targeted interventions based on the screening results holds promise for improving mental health outcomes and preventing suicide [101].

### Conclusions

The study’s timeline unfolds as follows. The data collection, which includes recruiting participants, conducting surveys, and gathering Facebook data, is scheduled to conclude by November 2024. Anticipating the system’s ability to accurately detect signs of depression in text and use patterns, we aim to advance early detection systems or interventions, completing the ML model by January 2025. The final results are expected to be available in February 2025.

By delving into these multifaceted aspects, this study will offer a comprehensive and pioneering approach to the field of mental health. Our approach to predicting depression through SM activity, or the examination, is not intended to replace conventional surveillance systems or laboratory-based depression diagnoses. To maximize the advantages of these approaches and concepts and improve people’s quality of life, we believe it is important to highlight the possibilities. This will also encourage discussion and raise awareness of any potential issues that should be resolved on individual and societal levels.

## Data Availability

The data set associated with this study is currently being collected and will be made available upon the completion of the data collection phase. A data availability statement will be provided once the data set is available for sharing.

## Abbreviations

API: application programming interface
LIWC: Linguistic Inquiry and Word Count
LMIC: low- or middle-income country
MDD: major depressive disorder
MI: mutual information
ML: machine learning
NLP: natural language processing
PHQ-9: 9-item Patient Health Questionnaire
PVP: problem video game playing
SM: social media
WGT: weekly gaming time
